# Effects of *PmDOME* and *PmSTAT* knockdown on white spot syndrome virus infection in *Penaeus monodon*

**DOI:** 10.1038/s41598-023-37085-1

**Published:** 2023-06-17

**Authors:** Pasunee Laohawutthichai, Thapanan Jatuyosporn, Premruethai Supungul, Anchalee Tassanakajon, Kuakarun Krusong

**Affiliations:** 1 Center of Excellence in Structural and Computational Biology, Department of Biochemistry, Faculty of Science, Chulalongkorn University, Bangkok, 10330 Thailand; 2 Center of Excellence for Molecular Biology and Genomics of Shrimp, Department of Biochemistry, Faculty of Science, Chulalongkorn University, Bangkok, 10330 Thailand; 3 National Center for Genetic Engineering and Biotechnology (BIOTEC), National Science and Technology Development Agency (NSTDA), Pathumthani, 12120 Thailand

**Keywords:** Innate immunity, Viral host response

## Abstract

Janus kinase/signal transducers and activators of transcription (JAK/STAT) signaling pathway plays an important role in antiviral immunity. This research reports the full-length *DOME* receptor gene in *Penaeus monodon* (*PmDOME*) and examines the effects of *PmDOME* and *PmSTAT* silencing on immune-related gene expressions in shrimp hemocytes during white spot syndrome virus (WSSV) infection. *PmDOME* and *PmSTAT* were up-regulated in shrimp hemocytes upon WSSV infection. Suppression of *PmDOME* and *PmSTAT* showed significant impacts on the expression levels of *ProPO2* (melanization), *Vago5* (interferon-like protein) and several antimicrobial peptides, including *ALFPm3, Penaeidin3, CrustinPm1* and *CrustinPm7*. Silencing of *PmDOME* and *PmSTAT* reduced WSSV copy numbers and delayed the cumulative mortality caused by WSSV. We postulated that suppression of the JAK/STAT signaling pathway may activate the proPO, IFN-like antiviral cytokine and AMP production, resulting in a delay of WSSV-related mortality.

## Introduction

*Penaeus monodon* or black tiger shrimp is one of the most invaluable shrimp in the aquaculture industry contributing to economic prosperity. However, over the past years, the amount of shrimp export has reduced, mainly due to viral disease outbreaks. White spot syndrome virus (WSSV) is a major virus pathogen causing white spot syndrome and accounted for almost 100% mortality within a few weeks after infection^1^. So far, there is no proven method for 100% preventing WSSV outbreaks. When foreign particles or pattern pathogen-associated molecular patterns (PAMPs) attach to pattern recognition proteins (PRPs) or receptors (PRRs) in shrimp, the innate immune system is activated through intracellular signaling cascades. The humoral immune responses include the melanin synthesis by the prophenoloxidase (proPO) system, blood clotting system and the generation of circulating antimicrobial peptides (AMPs)^2^. The cellular responses, on the other hand, cause apoptosis, phagocytosis, nodule formation and encapsulation^3^.

The Janus kinase/signal transducers and activators of transcription (JAK/STAT) pathway was first identified in mammalian systems and shown to transduce the various cytokines and growth factor signals^4^. The JAK/STAT pathway plays a role in haematopoiesis, immune function, cell growth, differentiation and development^5^ as well as innate immune and adaptive immune systems^6^. In mammalian, the main components of the JAK/STAT pathway are wide and diverse range of extracellular ligands (i.e. growth factors, interferons, interleukins and colony-stimulating factors), transmembrane receptors (i.e. interferon receptors IFNAR1, IFNAR2, IFNGR1 and IFNGR2), four JAK members (JAK1, JAK2, JAK3 and TYK2) and seven STAT proteins (STAT1, STAT2, STAT3, STAT4, STAT5A, STAT5B and STAT6)^7^. On the contrary, the JAK/STAT pathway of invertebrates is simpler than vertebrates with only a few components necessary for the signal transduction^8^. In *Drosophila*, the JAK/STAT pathway consists of three unpaired ligands (UPD1, UPD2 and UPD3)^9^, a receptor (Domeless or DOME)^10^, a JAK (Hopscotch or HOP)^11^ and a STAT (STAT92E)^12^. The binding of ligand to the Domeless receptor activates the receptor associated JAK tyrosine kinase Hopscotch, leading to phosphorylation of the receptor/JAK complex, which subsequently phosphorylates STAT92E dimer. Once the activated STAT92E dimers are translocated into the nucleus, they can bind to consensus DNA recognition motifs in the promoters, resulting in a transcription activation^13^. Several genes including *TotA*, *TotM* and virus induced RNA-1 (*vir-1*) contain STAT-binding sites in their promoter and are activated by the JAK/STAT pathway. Moreover, multiple regulatory proteins have been reported in *Drosophila* such as protein inhibitor of activated STAT (PIAS), protein tyrosine phosphatases (PTPs) and the suppressor of cytokine signaling (SOCS), the gene expression of which is activated by STAT and thus creates feedback inhibition of the pathway^14^.

In Pacific white shrimp *Litopenaeus vannamei*, *Lv*DOMELESS enhanced the wsv069 promoter activity by acting on the STAT-binding motif and knocking down the *LvDOMELESS,* resulting in lower cumulative mortality of shrimp and less WSSV copies^15^. Meanwhile, *LvJAK* silencing caused a higher mortality rate and increased the WSSV viral load^16^. It was reported that shrimp STAT was hijacked by WSSV to enhance the viral immediate early gene expression^17^.

In this work, we report the full length of Domeless receptor in the black tiger shrimp *Penaeus monodon* (*Pm*DOME) and investigate the role of the JAK/STAT pathway during WSSV infection. RNA interference techniques and mortality study were employed and the transcription levels of genes in the Toll, Immune Deficiency (Imd), JAK/STAT, interferon regulatory factor (IRF)-Vago, proPO and AMPs were examined under the silencing of *PmDOME* or *PmSTAT* during WSSV challenge. This work might lead to a better understanding of the characteristic of *PmDOME* and the relationship between the JAK/STAT pathway and the immune-related genes in response to WSSV infection in the black tiger shrimp.

## Results

### Cloning and sequence analysis of *Pm*DOME

**Figure 1:**
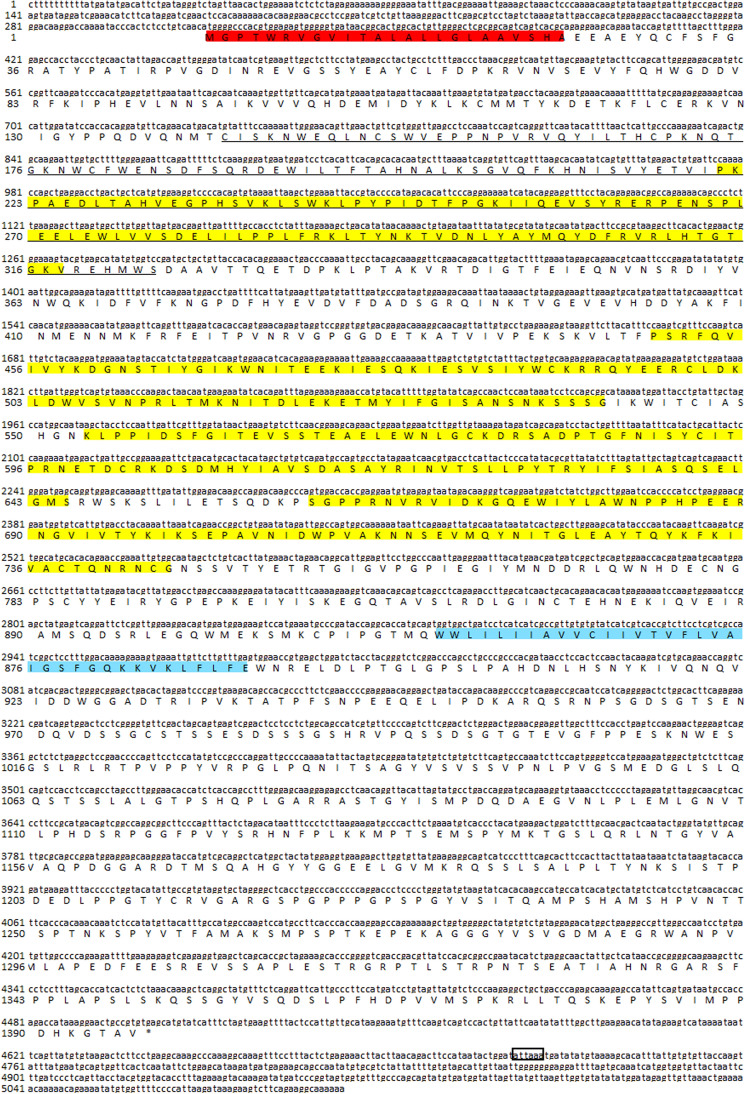
Nucleotide and deduced amino acid sequence of *Pm*DOME. Nucleotides and amino acids are both numbered on the left of the sequence. The open reading frame (ORF) of the nucleotide sequence is shown in upper-case letters, while the 5' and 3' UTRs are in lower-case letters. The signal peptide is shaded in red and the cytokine binding motif is underlined. The fibronectin-type-III like domains are highlighted in yellow. The transmembrane domain is in blue. The poly (A) signal is in the box.

The partial nucleotide sequence related to *Pm*DOME (2038 bp) was retrieved from the *P. monodon* EST database. The complete full length of *Pm*DOME was obtained by PCR amplification using specific primers (Supplementary Table S1) and a cDNA template prepared from healthy shrimp hemocytes. The full length of *PmDOME* is 5102 bp including 314 bp of the 5′-untranslated region (*5′-UTR*), 597 bp of *3′-UTR* containing a polyA signal sequence (ATTAAA) and 4191 bp of the open reading frame (ORF) that encoded a putative protein of 1396-amino acid residues (Fig. 1). The cDNA sequence was deposited in the NCBI database (MW187497). The calculated molecular weight of *Pm*DOME is 156.05 kDa and the isoelectric point (pI) is 5.81. The ORF of *Pm*DOME shared high sequence identity with Pacific whiteleg shrimp *Lv*DOMELESS (AGY46351.1, 94.70%)^15^ and DOME from *Marsupenaeus japonicus*, *Mj*DOME (APA16577.1, 87.12%)^18^. Meanwhile, *Pm*DOME exhibited 21.92% sequence identity with *Drosophila melanogaster* domeless (*Dm*DOME, NP_523412.1) and 28.57% sequence identity with human cytokine receptor (CAA41231.1).

**Figure 2:**
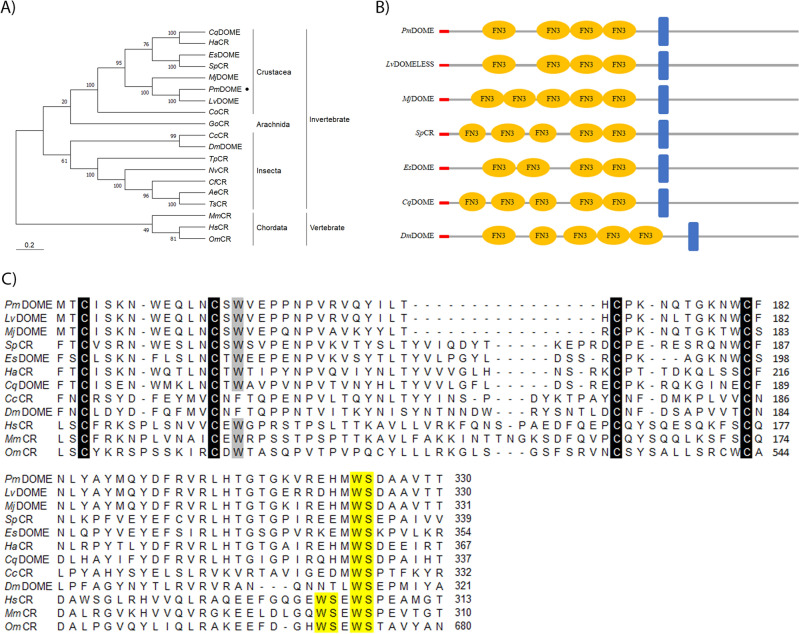
Sequence analysis of *Pm*DOME. (**A**) Phylogenetic tree of cytokine receptors from invertebrates and vertebrates. *Pm*DOME is shown with a dot. Analyzed proteins are *Cq*DOME, *Cherax quadricarinatus* (Accession No. QKU38106.1), *Ha*CR, *Homarus americanus* (Accession No. XP_042241219.1), *Es*DOME, *Eriocheir sinensis* (Accession No. QBA18592.1), *Sp*CR, *Scylla paramamosain* (Accession No. AHH29324.1), *Mj*DOME, *Marsupenaeus japonicus* (Accession No. APA16577.1), *Pm*DOME, *Penaeus monodon* (Accession No. MW187497), *Lv*DOME, *Litopenaeus vannamei* (Accession No. AGY46351.1), *Co*CR, *Chionoecetes opilio* (Accession No. KAG0716771.1), *Go*CR, *Galendromus occidentalis* (Accession No. XP_003744080.1), *Cc*CR, *Cyphomyrmex costatus* (Accession No. XP_004518381.1), *Dm*DOME, *Drosophila melanogaster* (Accession No. NP_523412.1), *Tp*CR, *Thrips palmi* (Accession No. XP_034251704.1), *Nv*CR, *Nasonia vitripennis* (Accession No. XP_008202634.1), *Cf*CR *Camponotus floridanus* (Accession No. XP_ EFN69830.1), *Ae*CR *Acromyrmex echinatior* (Accession No. EGI63440.1), *Ts*CR *Trachymyrmex septentrionalis* (Accession No. KYN30898.1), *Mm*CR *Mus musculus* (Accession No. CAA37810.1), *Hs*CR *Homo sapiens* (Accession No. CAA41231.1), *Om*CR *Oncorhynchus mykiss* (Accession No NP_001268335.1). CR indicates a cytokine receptor. (**B**) Domain diagrams of *Pm*DOME and cytokine receptors from various species. Regions shaded in red refer to signal peptides. Fibronectin type III like domains and transmembrane regions are shown in yellow and blue, respectively. (**C**) Multiple sequence alignment of the N-terminal of cytokine receptors. Four conserved cysteines and the tryptophane in a CX-_(9–10)_-CXWX-_(26–32)_-CX-_(10–15)_-C sequence are highlighted in black and gray, respectively. WSXWS motif is present in a yellow box.

Moreover, the phylogenetic tree was performed in MEGA 11.0 software to construct a neighbor-joining (NJ) phylogenetic tree based on the deduced amino acid sequence. The bootstrap sampling was reiterated 1000 times and demonstrated that *Pm*DOME was clustered into the group of invertebrates and closely related to those from *L. vannamei* and *M. japonicus* (Fig. 2A)*.* NetNGlyc-1.0 and NetOGlyc-4.0 servers predicted that *Pm*DOME contains 19 possible N-glycosylation sites based on the presence of the N-Xaa-T/S motif, while there are 15 possible O-glycosylation sites in the full-length of *Pm*DOME. Meanwhile, the GlycoEP server estimated 26 potential N-glycosylation sites and 59 O-glycosylation sites for *Pm*DOME.

From protein motif prediction, *Pm*DOME possessed 24 residues of a signal peptide, a cytokine binding motif, four fibronectin type III (FNIII) domains and a transmembrane domain. Figure 2B demonstrates domain diagrams of *Pm*DOME and others from seven closely related species. *Pm*DOME shows four FNIII domains, similar to Pacific whiteleg shrimp *Lv*DOMELESS and Chinese mitten crab *Es*DOME. In contrast, kuruma shrimp *Mj*DOME displayed five FNIII domains, similar to redclaw crayfish *Cherax quadricarinatus* DOME (*Cq*DOME), *Dm*DOME and cytokine receptor from mud crab *Scylla paramamosain* (*Sp*CR). Previously, *Dm*DOME was shown most similarities to the interleukin-6 (IL-6) receptor family^10^. In vertebrates, IL-6 receptors belong to the type I cytokine receptor family, identified by the cytokine binding homology region (CHR). CHR possesses four conserved cysteine residues arranged in a CX-_(9–10)_-CXWX-_(26–32)_-CX-_(10–15)_-C sequence, forming two disulfide bonds at the N-terminal domain and a WSXWS motif at the C-terminal domain^19^. In this research, the amino acid sequences of *Pm*DOME and cytokine receptors from 11 species were analyzed using the Clustal Omega tool. Multiple sequence alignment showed that *Pm*DOME contained four cysteine residues at the N-terminal domain and an incomplete WSXWS motif at the C-terminus, similar to other invertebrate cytokine receptors. Meanwhile, the vertebrate cytokine receptors, *Hs*CR, *Mm*CR and *Om*CR showed four conserved cysteine residues, along with a full WSXWS motif (Fig. 2C). It is worth noting that while *Pm*DOME and other examined cytokine receptors from both invertebrates and vertebrates contain a tryptophane in a CX-_(9–10)_-CXWX-_(26–32)_-CX-_(10–15)_-C sequence, phenylalanine is present in this particular sequence of *Cc*CR and *Dm*DOME. Based on sequence analysis, *Pm*DOME shared several characteristics with the vertebrate cytokine class I receptors and invertebrate DOME receptors, suggesting that *Pm*DOME is a signal-transducing receptor, involved in the JAK/STAT pathway.

### Tissue distribution of *PmDOME, PmJAK* and *PmSTAT* transcripts in shrimp

**Figure 3:**
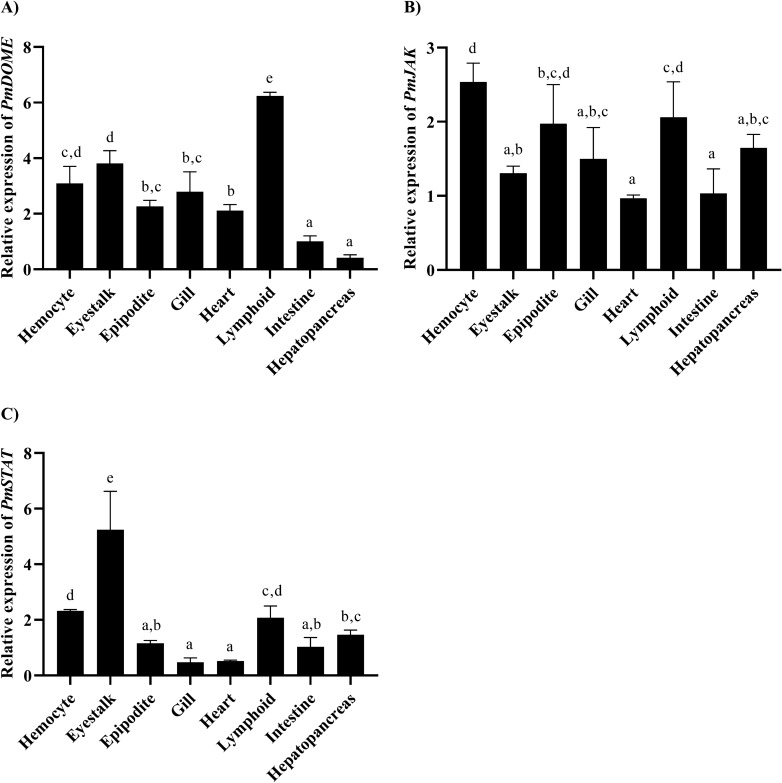
Tissue distribution of *PmDOME*, *PmJAK* and *PmSTAT* in healthy *P. monodon*. Expression profiles of *PmDOME* (**A**), *PmJAK* (**B**) or *PmSTAT* (**C**) in shrimp. The expression level in the intestine was used as a control and set as 1.0. Bars indicate the mean ± SD. Statistical analysis was performed using one-way ANOVA followed by Duncan’s new multiple range test. The experiment was carried out in triplicate and considered with different letters for statistical differences with significance at *p* < 0.05.

The distributions of *PmDOME, PmJAK* and *PmSTAT* transcripts in various tissues of healthy shrimp were determined by quantitative RT-PCR using the elongation factor-1α gene (*EF-1α*) as an internal control. *PmDOME* was expressed at the highest level in lymphoid, followed by eyestalk, hemocyte and gill, respectively, while the lowest expression level of *PmDOME* was found in hepatopancreas (Fig. 3A). The highest level of *PmJAK* transcript was found in hemocyte, followed by lymphoid and epipodite, respectively (Fig. 3B). The heart and intestine were the organs that contained the lowest level of *PmJAK* transcript among the test tissues. Moreover, the highest expression of *PmSTAT* was detected in eyestalk, followed by hemocyte and lymphoid, respectively. The lowest level of *Pm*S*TAT* transcript was in gill and heart (Fig. 3C).

### Expression profiles of *PmDOME* and *PmSTAT* in response to WSSV infection and their dsRNA efficiency

In this study, the RNA interference (RNAi) technique was used to knock down *PmDOME* and *PmSTAT* transcripts in order to investigate the role of the JAK/STAT pathway in shrimp’s innate immune system. *PmDOME* dsRNA, *PmSTAT* dsRNA and *GFP* dsRNA were successfully synthesized with the size of approximately 500 bp, 250 bp and 700 bp, respectively. Shrimp were injected with either 5 or 10 µg of *PmDOME* dsRNA per 1 g shrimp. Injection of 5 µg of *PmDOME* dsRNA per 1 g of shrimp could knockdown *PmDOME* gene expression by approximately 66% at 24 hpi, whereas injection of 10 µg of *PmDOME* dsRNA per 1 g of shrimp could reduce *PmDOME* gene expression by about 50% at 24 hpi (Supplementary data, Fig. S1A). Meanwhile, shrimp were received a double injection of either 10 µg + 5 µg *PmSTAT* dsRNA per 1 g shrimp or 10 µg + 10 µg *PmSTAT* dsRNA per 1 g shrimp. A set of double injection of 10 µg + 10 µg *PmSTAT* dsRNA per 1 g shrimp was more efficient than a set of 10 µg + 5 µg *PmSTAT* dsRNA per 1 g shrimp to lower  the *PmSTAT* gene expression (Supplementary data, Fig. S1B). Double injection of 10 µg + 10 µg *Pm*STAT dsRNA per 1 g shrimp decreased *PmSTAT* transcript by approximately 79% at 24 hpi. As a result, dosages of 5 µg of *PmDOME* dsRNA per 1 g shrimp and double injection of 10 µg + 10 µg *PmSTAT* dsRNA per 1 g shrimp were used to knock down the *PmDOME* and *PmSTAT* expression, respectively.

**Figure 4:**
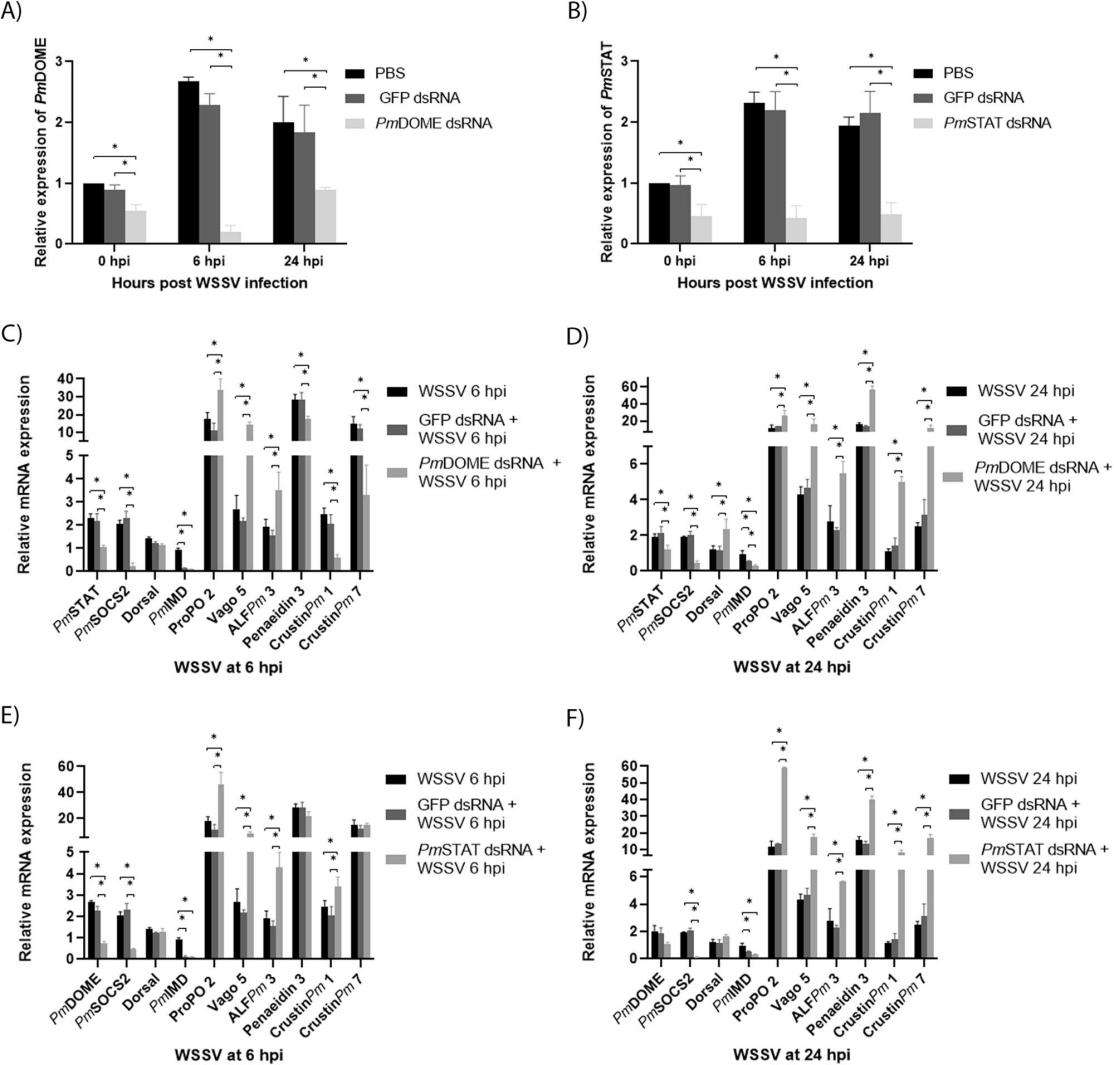
Effects of *PmDOME* and *PmSTAT* knockdown in *P. monodon*. Expression profiles of *PmDOME* (**A**) or *PmSTAT* (**B**) in shrimp after either *PmDOME* or *PmSTAT* dsRNA injection and WSSV challenge at 6 and 24 hpi. The gene expression levels were relative to that of the control (PBS groups, in which the gene expression level was set to 1.0 at 0 hpi). Transcription levels of immune-related genes in *PmDOME* (**C**,**D**) and *PmSTAT* (**E**,**F**) silenced shrimp at 6 h and 24 h after WSSV infection. Bars indicate the mean ± SD. Statistical analysis was performed using one-way ANOVA followed by Duncan’s new multiple range test. The data were derived from independently triplicate experiments and considered for statistical differences with significance at *p* < 0.05.

As shown in Fig. 4A,B, WSSV infection caused an increase in *PmDOME* and *PmSTAT* expression levels. *PmDOME* mRNA levels in control and *GFP* dsRNA treated groups were up-regulated around 2.5- and 2-fold at 6 hpi and 24 hpi, respectively, in comparison to the unchallenged group. Similarly, *PmSTAT* transcription levels in control and *GFP* dsRNA injected shrimp increased by twofold at 6 h and 24 h after WSSV challenge. Although WSSV infection seemed to stimulate *PmDOME* and *PmSTAT* expression, the transcript levels of these genes remained low in the *PmDOME* and *PmSTAT* knockdown shrimp upon WSSV infection.

### Effects of *PmDOME* and *PmSTAT* silencing on immune-related genes during WSSV infection

To investigate the role of the JAK/STAT signaling pathway during WSSV infection, the mRNA levels of immune-related genes in *PmDOME* and *PmSTAT* knockdown shrimp were determined by qRT-PCR genes. These included genes in the signaling pathways (*PmDorsal*, *PmIMD* and *PmSOCS2*), melanization process (*ProPO2*), IFN-like gene (*PmVago5*) and AMPs (*ALFPm3, Penaeidin3, CrustinPm1* and *CrustinPm7*). As shown in Fig. 4C–F, expressions of *ProPO2, Penaeidin3* and *CrustinPm7* expressions significantly increased by 17, 28 and 14-fold at 6 h and 12, 16 and 2.5-fold at 24 h after WSSV challenge, in comparison with unchallenged shrimp. This suggested that *ProPO2, Penaeidin3* and *CrustinPm7* play an important role in WSSV defense. Other genes including *PmSTAT, PmDOME, PmSOCS2*, *Vago5* and *ALFPm3* were also up-regulated (> twofold) at 6 and 24 hpi. It is worth mentioning that *Pm*Dorsal and *PmIMD* expression levels, representing the Toll and Imd pathway, respectively, were insignificantly changed at 6 and 24 hpi. In addition, *CrustinPm1* was up-regulated by 2.5-fold at 6 hpi but its transcript level was not substantially different at 24 hpi, compared with that of the control group.

*PmDOME*-deprived shrimp also showed down regulation of *PmSTAT* and *PmSOCS2* at 6 and 24 hpi (Fig. 4C,D). *PmDOME* silenced shrimp exhibited much higher levels of *ProPO2*, *Vago5* and *ALFPm3* than that in control and *GFP* knockdown shrimp upon WSSV infection. As shown in Fig. 4D, *ProPO2*, *Vago5* and *ALFPm3* in *PmDOME* silenced shrimp was up-regulated by 26, 16 and 5-fold at 24 hpi, compared with the control group. Suppression of *PmDOME* lowered the expression of *Penaeidin3*, *CrustinPm1* and *CrustinPm7* at 6 hpi but significantly increased these gene transcripts at 24 hpi, compared with that in control and *GFP*-treated shrimp infected by WSSV. Notably, the expressions of *Penaeidin3*, *CrustinPm1* and *CrustinPm7* in *PmDOME*-depleted shrimp increased by 57, 5 and 12-fold at 24 h after WSSV challenge.

Consistent with *PmDOME* silencing, the knockdown of *PmSTAT* also lower expressions of *PmDOME* and *PmSOCS2* (Fig. 4E,F). Suppression of *PmSTAT* also led to an increase of *ProPO2*, *Vago5* and *ALFPm3* at 6 and 24 hpi. As illustrated in Fig. 4F, the expression levels of *ProPO2*, *Vago5* and *ALFPm3* in *PmSTAT* silenced shrimp were increased by 59, 17.5 and 5.6-fold, respectively at 24 hpi. *Penaeidin3*, *CrustinPm1* and *CrustinPm7* transcripts were found to be lower in *PmSTAT*-silenced shrimp at 6 hpi, compared with that of WSSV-infected control and *GFP*-treated shrimp. Similar to *PmDOME* knockdown shrimp, *PmSTAT* depleted shrimp showed an increase of *Penaeidin3*, *CrustinPm1* and *CrustinPm7* at 24 hpi by 40, 8 and 17-fold, compared with that of unchallenged shrimp. Clearly, *PmDOME* and *PmSTAT* silencings gave similar results and the JAK/STAT signaling pathway significantly affected *ProPO2* (melanization), *Vago5* (interferon-like protein) and *AMP* gene expressions.

### Survival rate of WSSV-infected shrimp and viral copy number after *PmDOME* and *PmSTAT* silencing

**Figure 5:**
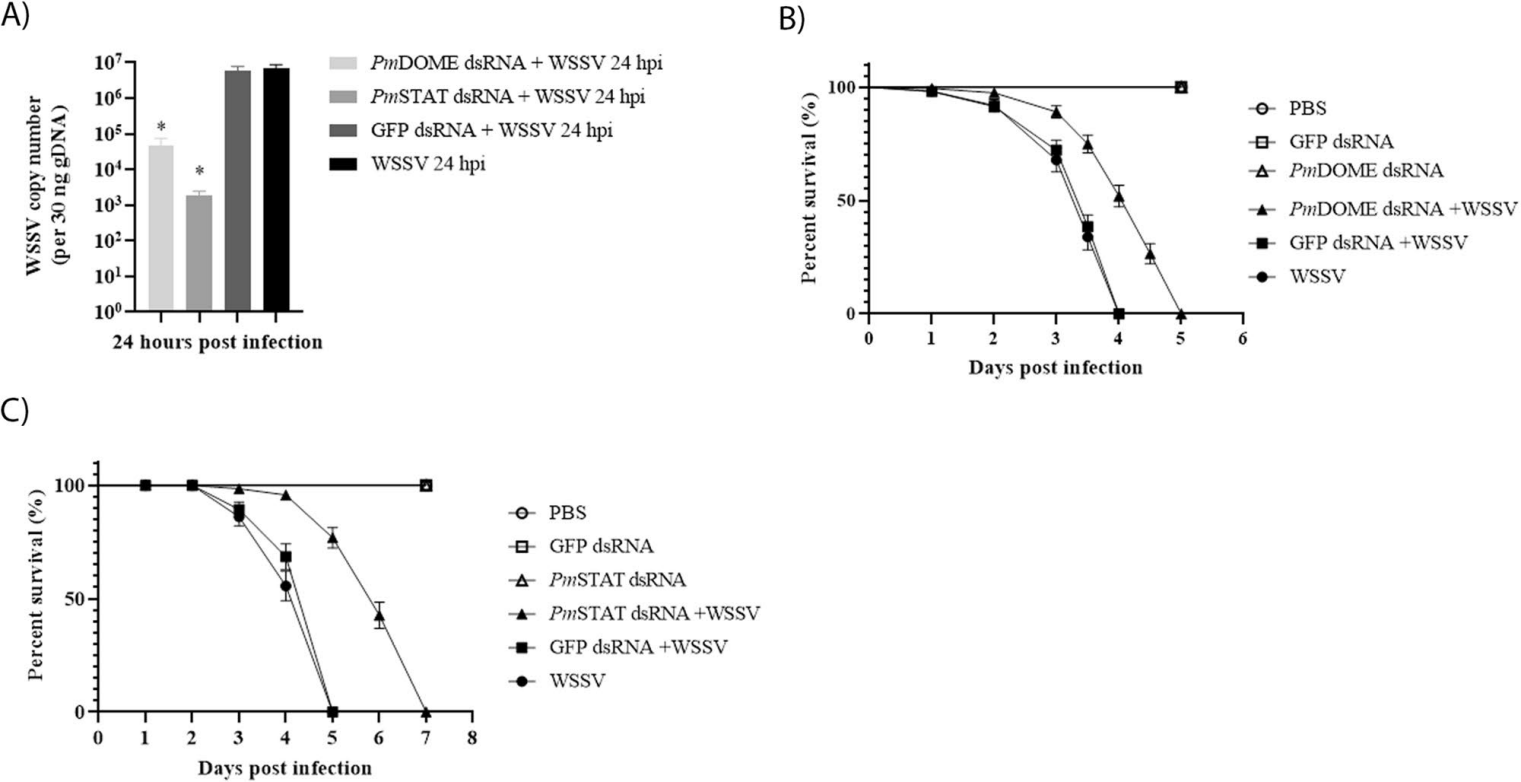
Effects of *PmDOME* and *PmSTAT* silencing during WSSV infection. (**A**) WSSV genome copies in gill tissues (30 µg) of *PmDOME*, *PmSTAT* and *GFP* dsRNA treated shrimps were measured at 24 hpi. Bars indicate the mean ± SD. Statistical analysis was performed using one-way ANOVA followed by Duncan’s new multiple range test. The data were derived from independently triplicate experiments and considered for statistical differences with significance at *p* < 0.05. (**B**,**C**) Cumulative mortality of *PmDOME* or *PmSTAT* silenced shrimp caused by WSSV infection. Differences in mortality level between treatments were analyzed by the log-rank (*p* values < 0.0001).

To examine functions of the JAK/STAT pathway during WSSV infection, WSSV copy numbers in *PmDOME* and *PmSTAT* silenced shrimp were quantified by the detection of conserved *VP28* gene using qRT-PCR, in comparison with those in WSSV infected control and *GFP* treated group. Clearly, WSSV copy numbers in *PmDOME* and *PmSTAT* silenced shrimp were significantly lower than that in control and *GFP* treated shrimp (Fig. 5A). Moreover, knockdown of *PmDOME* and *PmSTAT* resulted in a delay of shrimp mortality (Fig. 5B,C). *PmDOME*-depleted shrimp reached 100% mortality on day 5 after WSSV infection, while *PmSTAT* knockdown shrimp attained 100% death on day 7.

## Discussion

Innate immune responses serve as the first line of defense against pathogen infections. These occur via signal transduction pathways to activate diverse humoral and cellular processes. The Toll, Imd and JAK/STAT signaling pathways play important roles in antiviral responses. In this study, we aim to investigate the functions of the JAK/STAT signaling pathway during WSSV infection.

In this research, the complete ORF of *Pm*DOME shared high sequence identities with previously reported Pacific whiteleg shrimp *Lv*DOMLESS^15^ and kuruma shrimp *Mj*DOME^18^, respectively. The phylogenetic tree revealed that the *Pm*DOME was clustered into the invertebrate group (Crustacea, Arachnida and Insecta) (Fig. 2A). Although the sequence of DOME differed between species, the main functional domains were relatively similar, including the presence of a signal peptide, several fibronectins type III like motifs, a transmembrane domain and the cytokine binding motif which involved in receptor activation^20^. *Pm*DOME belongs to the crustacean group and is most closely related to *Lv*DOMELESS, followed by *Mj*DOME*. Pm*DOME and *Lv*DOMELESS possess four fibronectin type III like domains, while *Mj*DOME has five fibronectin type III like domains and is similar to *Dm*DOME, the first identified invertebrate interleukin JAK/STAT receptor^10^ (Fig. 2B). *Pm*DOME might belong to the IL-6 like receptor family, which bind to extracellular cytokines and trigger intracellular signals. Its biological functions are considered similar to DOME from other crustaceans.

Since invertebrates lack adaptive immune response, hemocyte is important for innate immune response to eliminate the invading pathogens^21^. From tissue distribution, both *PmDOME* and *PmSTAT* transcript levels were high in the hemocyte (Fig. 3). As a result, the hemocyte was selected as a target to study the influence of *PmDOME* and *PmSTAT* knockdown upon WSSV infection.

As shown in Fig. 4, the JAK/STAT signaling pathway well responded to WSSV infection as *PmDOME*, *PmSTAT* and *PmSOCS2* expression levels increased (< twofold) at 6 and 24 hpi, while the mRNA levels of *Dorsal* and *PmIMD*, representing the Toll and IMD pathway, in unchallenged and WSSV-infected shrimp showed slight differences. In addition, the gene expressions of *ProPO2*, *Vago5*, *ALFPm3*, *Penaeidin3* and *CrustinPm7* were up-regulated at 6 and 24 hpi in WSSV-infected shrimp, compared with unchallenged shrimp (Fig. 4C–F). Meanwhile, the gene expression of *CrustinPm1* also increased at 6 hpi, but then dropped at 24 hpi. This is in agreement with previous reports showing that ALF*Pm*3^21^, Penaeidin3^22^, Vago^23,24^ and proPO system^25^ acted against WSSV.

Surprisingly, knockdown of either *PmDOME* or *PmSTAT* resulted in lower expression of the JAK/STAT components, including *PmDOME*, *PmSTAT* and *PmSOCS2*, but significantly enhanced *ProPO2, Vago5* and *ALFPm3* at 6 and 24 hpi (Fig. 4C–F). In addition, the expression of *Penaeidin3*, *CrustinPm1* and *CrustinPm7* in *PmDOME* or *PmSTAT*-deprived shrimp was also dramatically increased at 24 hpi (Fig. 4D,F). It is evident that knockdown of the JAK/STAT signaling pathway could enhance the proPO system, IFN-like antiviral cytokine and AMPs in response to WSSV infection. In addition, both *PmDOME* and *PmSTAT*-silenced shrimp showed lower WSSV copy numbers, compared with control and ds*GFP*-treated shrimp (Fig. 5A). This result reflects the fact that knockdown of *PmDOME* or *PmSTAT* delayed shrimp mortality caused by WSSV (Fig. 5B,C). In previous research, *CqDOME*-silenced hematopoietic tissue exhibited lower transcription levels of WSSV-immediately early gene (*IE1*) and a late gene envelope protein, VP28^26^. Suppression of *CqDOME* also decreased *Cq*STAT phosphorylation, which is required for activation of the *IE1* transcript^17^.

The proPO system, involved in the production of superoxides and hydroxyl radicals to kill the invading pathogen, is important in immune defense against WSSV infection^27^. For example, in red swamp crayfish *Procambarus clarkia*, injection with the recombinant proPO2 could significantly decrease 65% mortality of WSSV-infected crayfish and reduced the amount of WSSV copy number in hepatopancreas and gill^28^. In addition, the silencing of the *proPO* gene in freshwater prawn *Macrobrachium rosenbergi* makes them susceptible to WSSV^29^. It was reported that the *proPO* gene was highly up-regulated after LPS stimulation, while the lack of *LvSTAT* down-regulated the *proPO* expression. It was speculated that the JAK/STAT pathway activated the proPO system by controlling the differentiation of hemocytes to promote the humoral immune response^30^. In this research, *PmDOME* and *PmSTAT* silencing significantly enhanced the expression levels of *proPO2* by 30–60-fold during WSSV infection (Fig. 4C–F). This massive activation of the proPO system in *PmDOME* or *PmSTAT*-deprived shrimp may reduce WSSV infection and delay shrimp mortality.

It was reported that the interferon (*IFN*) regulatory factor (*IRF*)-like gene identified in *L. vannamei* could be activated by WSSV and bind the 5′-untranslated region of *L. vannamei Vago4* gene^31^. This suggested that IRF-Vago-JAK/STAT might exist in invertebrates, similar to the vertebrates IRF-IFN-JAK/STAT. Suppression of *LvVago 4/5* led to an increase in shrimp mortality by WSSV. Obviously, this result is consistent with our research, showing that *Vago5* expression was elevated when the JAK/STAT signaling pathway was disrupted (Fig. 4C–F) and perhaps delaying the effects of WSSV on shrimp mortality (Fig. 5B,C). It was shown previously that cholesta-2,5-diene, a lipid of WSSV envelope, was recognized by the lipid-recognition protein, ML1, in *M. japonicus*, resulting in Dorsal translocation into the nucleus and Vago expression^23^. In our work, suppression of *PmDOME* or *PmSTAT* did not alter the *Dorsal* transcription level, although the *Vago5* expression was enhanced by 8- to 17-fold at 6 and 24 hpi (Fig. 4C–F). It is possible that *Vago5* could be activated via alternative pathways. Li and co-workers reported that linoleic acid promoted the expressions of Vago5 and AMPs via ERK-NF-κB against WSSV^24^.

The elevated levels of AMP expressions in *PmDOME* and *PmSTAT*-silenced shrimp may also contribute to delayed mortality. Previously, ALF*Pm*3 has shown anti-WSSV activity via binding to the viral structural proteins and destroying the viral envelope^32,33^. In *L. vannamei*, silencing of the *Penaeidin* family including *BigPEN, PEN2, PEN3* and *PEN4* resulted in increasing cumulative mortality and WSSV copy numbers, while co-incubation of each recombinant penaeidin with WSSV inhibited the viral internalization into hemocytes^22^. It was demonstrated that PEN2 competitively bound to the envelope protein VP24, while BigPEN bound to VP28, which could disrupt WSSV infection^22^. Crustin*Pm*1 and Crustin*Pm*7 showed strong antimicrobial activity by agglutinating bacterial cells to disrupt the physiochemical properties of bacterial surface^34^. Although the antibacterial activity of crustin in shrimp is well known, its function in the viral infection process has been less well-studied. Recently, Zhang and co-workers reported that knockdown of *MnCRU1, MnCRU6* and *MnALF1* in the oriental river prawn *Macrobrachium nipponenese* resulted in increasing *VP28* expression and number of WSSV virions, indicating that crustin and ALF may play anti-WSSV roles in shrimp^35^.

It was previously reported that *ALFPm3*, *Penaeidin3* and *CrustinPm7* were regulated by Toll and Imd pathway^36,37^, whereas *CrustinPm1* was regulated by Toll pathway^38^. In this research, *PmDOME* and *PmSTAT* silencing did not affect the expression of *PmDorsal* and *PmIMD,* representing the Toll and Imd pathway. We hypothesized that the *Vago5* expression was significantly increased in *PmDOME* and *PmSTAT*-silenced shrimp, in order to compensate for the downregulation of the JAK/STAT signaling pathway, resulting in the enhancement of AMP production. In kuruma shrimp, *Mj*Vago-L was reported to activate the JAK/STAT and induce the downstream effector *Mj*Ficolin through integrin during WSSV infection^39^.

In previous work, we reported that WSSV caused a decrease in the richness and diversity of microbiota^40^. In addition, WSSV-challenged shrimp showed higher levels of *Photobacterium damselae*, a pathogenic marine bacterium, while *Shewanella algae*, a shrimp probiotic, was reduced. Since the microbiota of *PmSTAT*-silenced shrimp was different from that of the control, *STAT* might function to maintain host-microbiota interactions. Taken together, the JAK/STAT signaling pathway response to WSSV infection via regulation of immune-related gene expressions and microbial homeostasis.

## Methods

### Shrimp

Healthy black tiger shrimp (*Penaeus monodon)* of average 3 g body weight were obtained from a shrimp farm in Chachoengsao Province, Thailand. Shrimp were acclimated in recirculating water tank system filled with air-pumped seawater with a salinity of 20 ppt at the temperature of 28 ± 4 °C. They were fed with a commercial diet twice a day for at least 1 week before the experiments. This study was conducted under the ethical principles and guidelines according to the animal use protocol 1923021 approved by Chulalongkorn University Animal Care and Use Committee (CU-ACUC). The biosafety guidelines were reviewed and approved by the Institutional Biosafety Committee of Chulalongkorn University (SC-CU-IBC-004-2018).

### Cloning of the full-length *PmDOME*

The ORF of *Pm*DOME was obtained from the hemocyte cDNA by PCR amplification using specific primers (Supplementary data, Table S1) based on a partial sequence of *PmDOME* (accession no. PM 89949) in the expressed sequence tag (EST) database of *P. monodon* (http://pmonodon.biotec.or.th)^3^. The full length of *PmDOME* gene was amplified from the hemocyte cDNA of *P. monodon* using the 5′ upstream region sequence (UPDOME-F1 primer) and 3′ downstream region sequence (DOME-R primer). The PCR conditions were as followed; (1) 96 °C for 5 min, (2) 25 cycles of 96 °C for 20 s, (3) 56 °C for 30 s, (4) 68 °C for 5 min, (5) a final extension at 68 °C for 7 min. The *PmDOME* PCR product was ligated into pGEX 6P-3 vector using an In-Fusion cloning kit (Takara) with the following conditions; 37 °C for 15 min, 50 °C for 15 min and 4 °C for 5 min; and then transformed into *Escherichia coli* TOP10 (Invitrogen). The recombinant plasmid named pGEX 6P-3-*PmDOME* was verified by sequencing. The full-length *PmDOME* gene was deposited in the NCBI GenBank (GenBank accession No. MW187497).

### Bioinformatics analysis of *Pm*DOME

The amino acid sequence of *Pm*DOME was determined by ExPASy-Translate tool (https://web.expasy.org/translate/) and the protein motif was analyzed by SMART (http://smart.embl-heidelberg.de/) and GenomeNet (https://www.genome.jp/tools/motif/). The polyadenylation site in *Pm*DOME sequences was predicted by Poly(A) Signal Miner (http://dnafsminer.bic.nus.edu.sg/PolyA.html). The predicted molecular weight of *Pm*DOME was calculated by Compute pI/MW (https://web.expasy.org/compute_pi). Clustal Omega program was used to analyze the multiple sequence alignments (https://www.ebi.ac.uk/Tools/msa/clustalo/). The phylogenetic analysis was performed using MEGA 11 software to construct a neighbor-joining phylogenetic tree based on the deduced amino acid sequences. The bootstrap sampling was reiterated 1000 times. The N- or O-glycosylation sites of *Pm*DOME was predicted by NetNGlyc-1.0^41^ or NetOGlyc-4.0^42^ and by GlycoEP server^43^.

### Preparation of WSSV stock

WSSV was prepared from the gill tissue of WSSV-infected moribund shrimp using ultracentrifugation and membrane filtration as described in Jaturontakul et al.^44^.

### Double strands RNA (dsRNA) synthesis

*PmDOME* dsRNA, *PmSTAT* dsRNA and *GFP* dsRNA were synthesized using T7 RiboMAX™ Express Large Scale RNA Production System (Promega). Two sets of specific primers each for *PmDOME*, *PmSTAT* and *GFP* genes were designed as shown in Supplementary Table S1. One of the specific primer pairs contained the T7 promoter sequence at the 5′ end. The hemocyte cDNA of *P. monodon* was used as a template to amplify *PmDOME* dsRNA and *PmSTAT* dsRNA, while the recombinant *GFP* plasmid was used as a template to amplify *GFP* dsRNA. The two PCR products each of *PmDOME*, *PmSTAT* and *GFP* genes were separately amplified by the specific primer pairs using the following conditions; 94 °C for 2 min, followed by 35 cycles of 98 °C for 10 s, 58 °C for 30 s and 68 °C for 30 s and a final extension at 68 °C for 7 min. Subsequently, the dsRNA of *PmDOME*, *PmSTAT* and *GFP* was produced in vitro using T7 RiboMAX™ Express Large Scale RNA Production System (Promega). The quality of synthesized dsRNAs was examined by 2% agarose gel electrophoresis and the concentration was determined by measuring absorbance at 260 nm and stored at − 80 °C for further use.

### Total RNA isolation and first-stranded cDNA synthesis

Tissue samples from healthy and pathogen-infected shrimp were collected and homogenized in FARB buffer (Tissue Total RNA mini kit, Favorgen, Taiwan). Total RNA was extracted by following the manufacturer’s protocol. Five hundred nanograms of total RNA were converted to the cDNA by RevertAid First Strand cDNA Synthesis Kit (Thermo Fisher, USA). The cDNA was kept at − 20 °C for further use.

### Analysis of *PmDOME*, *PmJAK* and *PmSTAT* gene expressions in shrimp

Shrimp tissues including hemocyte, eyestalk, epipodite, gill, heart, lymphoid, intestine and hepatopancreas were collected from 9 healthy shrimp and total RNA was extracted by Tissue Total RNA mini kit (Favorgen), followed by cDNA synthesis using the RevertAid First Strand cDNA Synthesis Kit (Thermo Fisher). *PmDOME*, *PmJAK* and *PmSTAT* gene expression levels in each tissue were identified by quantitative RT-PCR using 2 µL of tenfold diluted cDNA template and specific primers shown in Supplementary Table S1. Elongation factor-1α (*EF-1α*) gene was used as an internal control. The expression level in the intestine was used as a control and set as 1.0.

The quantitative RT-PCR (qRT-PCR) was carried out using an equal amount of cDNAs in the CFX96 Touch™ Real-Time PCR System and the Luna^®^ Universal qPCR Master Mix (NEB) in the following conditions: one cycle at 95 °C for 1 min, followed by 45 cycles of 95 °C for 15 s and 60 °C for 30 s, using specific primers as shown in Supplementary Table S1. The expression of the elongation factor-1α gene (*EF-1α*) was used as an internal control. Melt curve analysis was performed at the end of the PCR thermal cycle to determine the specificity of amplification. The relative expressions of *PmDOME* and *PmSTAT* were calculated using a comparative C_T_ method with the 2^−ΔΔC^_T_^45^. The data were shown as means ± standard deviations (SD). Statistical analysis was performed using one-way ANOVA followed by Duncan’s new multiple range test. The data were considered for statistical differences with the significance at *p* < 0.05.

### Effects of *PmDOME* and *PmSTAT* silencing on immune-related genes after WSSV infection

To verify the silencing efficiency of dsRNA in vivo*,* shrimp with an average body weight of 3 g were injected with different dosages of either *PmDOME* dsRNA or *PmSTAT* dsRNA through intramuscular injection. A single injection of *PmDOME* dsRNA contained either 5 or 10 µg dsRNA per 1 g shrimp. Meanwhile, either a set of 10 µg dsRNA, followed by 10 µg dsRNA or a set of 10 µg dsRNA, followed by 5 µg dsRNA per 1 g shrimp was used in a double injection of *PmSTAT* dsRNA with 24 h interval between each injection. The dsRNAs were dissolved in 1X-PBS, pH 7.4 and the experiments were carried out in triplicate. The control group was injected with 1 × PBS (pH 7.4). Three shrimp hemolymphs in each group were collected at 24 h post injection. The hemolymph was collected using a sterile 1-mL syringe with 500 μL of anticoagulant, pH 7.0 (27 mM sodium citrate, 336 mM sodium chloride, 115 mM glucose, 9 mM ethylenediaminetetraacetic (EDTA). The hemolymph-anticoagulant mixture was then centrifuged at 800×*g* for 15 min at 4 °C to separate the hemocytes from the plasma.

To examine the effects of *PmDOME* and *PmSTAT* silencing on immune-related genes upon WSSV infection, shrimp with an average body weight of 3 g were divided into ten groups with three shrimps per group. The experiment was carried out in triplicate. Shrimp in groups 1 and 2 were injected with 1 × PBS (pH 7.4), while shrimp in groups 3 and 4 were injected with 5 µg of *PmDOME* dsRNA per 1 g of shrimp; and shrimp in groups 5 and 6 received 5 µg of *GFP* dsRNA per 1 g of shrimp as a control of *PmDOME* silenced group. Shrimp in groups 7 and 8 were double injected with 10 µg of *PmSTAT* dsRNA per 1 g of shrimp and shrimp in groups 9 and 10 were double injected with 10 µg of *GFP* dsRNA per 1 g of shrimp as a control of  the *PmSTAT* silenced group. After 24 h post dsRNA injection, shrimp in the even-numbered groups were infected with ~ 6 × 10^6^ viral copies of WSSV. The hemolymph in each group was collected at different time points, 6 and 24 h post-WSSV infection, before the total RNA was extracted and converted to cDNA. The expression profiles of immune-related genes including *PmSOCS2*, *Dorsal*, *PmIMD*, *Vago5*, *ProPO2, ALFPm3*, *Penaeidin3, CrustinPm1* and *CrustinPm7* were determined by qRT-PCR using gene specific primers in Supplementary Table S1.

### Quantification of WSSV copy number in shrimp

To study the effect of *PmDOME* and *PmSTAT* suppression on WSSV replication, the WSSV copy number in shrimp was determined. The shrimp’s gill samples were collected in parallel with the WSSV challenge experiment. Shrimp genomic DNA was extracted using FavorPrep Tissue Genomic DNA Extraction Mini Kit (Favorgen, Taiwan) and quantified by NanoDrop™ 2000c Spectrophotometer (Thermo Scientific). The extracted sample was used as a DNA template for viral copy number analysis. qRT-PCR was performed in triplicate using Luna^®^ Universal qPCR Master Mix (NEB) with 2 μL genomic DNA (15 ng/μL) and *VP28* primers (Supplementary Table S1). The cycling condition was previously mentioned. The recombinant plasmid containing a conserved region of WSSV *VP28* gene was used to prepare the standard curve.

### Mortality assays of *PmDOME* and *PmSTAT* silencing shrimp upon WSSV infection

Shrimps were separated into six groups and each contained 10 shrimps per group. The assay was conducted in triplicate. Shrimp in groups 1 and 2 were injected with 1 × PBS (pH 7.4), while those in groups 3 and 4 were either single injected with 5 µg of *PmDOME* dsRNA per 1 g of shrimp or double injected with 10 µg of *PmSTAT* dsRNA per 1 g of shrimp. Groups 5 and 6 were control groups; and the animals were either single injected with 5 µg of *GFP* dsRNA per 1 g of shrimp or double injected with 10 µg of *GFP* dsRNA per 1 g of shrimp. Shrimp in the even-numbered group were then injected with ~ 6 × 10^6^ viral copies of WSSV at 24 h after dsRNA infection. The cumulative mortalities were recorded every 12 h after WSSV infection up to 7 days. The cumulative mortality experiment of *PmDOME* and *PmSTAT* silenced shrimp were performed separately. Data were analyzed using GraphPad Prism 8 and presented as percent survival with the *p* values (< 0.0001) calculated by the logrank test.

## Supplementary Information

Supplementary Information.

## Data Availability

All data generated or analyzed during this study are included in this published article and its supplementary information file.

## Abbreviations

ALF: Anti-lipopolysaccharide factor
AMPs: Antimicrobial peptides
IFN: Interferon
Imd: Immune deficiency
JAK/STAT: Janus kinase/signal transducers and activators of transcription
*Pm*: *Penaeus monodon*
proPO: Prophenoloxidase
WSSV: White spot syndrome virus
