# Supplementary material for: Effects of PmDOME and PmSTAT knockdown on white spot syndrome virus infection in Penaeus monodon
Source: Sci Rep. 2023 Jun 17;13:9852. doi: 10.1038/s41598-023-37085-1 (PMC10276838; doi:10.1038/s41598-023-37085-1)
Supplement: Supplementary file 1 — Supplementary Information. [file 41598_2023_37085_MOESM1_ESM.docx]

Effects of *PmDOME* and *PmSTAT* knockdown on white spot syndrome virus infection in *Penaeus monodon*

Pasunee Laohawutthichai^1,2^, Thapanan Jatuyosporn^1,2^, Premruethai Supungul^2,3^, Anchalee Tassanakajon^2^, Kuakarun Krusong^1,*^

^1^Center of Excellence in Structural and Computational Biology, Department of Biochemistry, Faculty of Science, Chulalongkorn University, Bangkok 10330, Thailand

^2^Center of Excellence for Molecular Biology and Genomics of Shrimp, Department of Biochemistry, Faculty of Science, Chulalongkorn University, Bangkok 10330, Thailand

^3^National Center for Genetic Engineering and Biotechnology (BIOTEC), National Science and Technology Development Agency (NSTDA), Pathumthani 12120, Thailand

*** Correspondence:**Kuakarun Krusong
[Kuakarun.k@chula.ac.th](mailto:Kuakarun.k@chula.ac.th)

**Supplementary data**

**Table S1** Nucleotide sequences of the primers

| Primer name | Sequence (5’ – 3’) | Experiment |
| --- | --- | --- |
| dsDOME-T7-F | GGATCCTAATACGACTCACTATAGG | double-stranded RNA synthesis |
|  | CAGTGGAACCACGATGAATG |  |
| dsDOME-F | CAGTGGAACCACGATGAATG | double-stranded RNA synthesis |
| dsDOME-T7-R | GGATCCTAATACGACTCACTATAGG | double-stranded RNA synthesis |
|  | GCACGATCTTGTAGTTGGAG |  |
| dsDOME-R | GCACGATCTTGTAGTTGGAG | double-stranded RNA synthesis |
| dsSTAT-T7-F | GGATCCTAATACGACTCACTATAGG | double-stranded RNA synthesis |
|  | GCCAGTTGTAGTCATTGTCC |  |
| dsSTAT-F | GCCAGTTGTAGTCATTGTCC | double-stranded RNA synthesis |
| dsSTAT-T7-R | GGATCCTAATACGACTCACTATAGG | double-stranded RNA synthesis |
|  | CAAAGCTGCCACTGGAAGGG |  |
| dsSTAT-R | CAAAGCTGCCACTGGAAGGG | double-stranded RNA synthesis |
| dsGFP-T7-F | GGATCCTAATACGACTCACTATAGG | double-stranded RNA synthesis |
|  | CAGTGCTTCAGCCGCTACCC |  |
| dsGFP-F | CAGTGCTTCAGCCGCTACCC | double-stranded RNA synthesis |
| dsGFP-T7-R | GGATCCTAATACGACTCACTATAGG | double-stranded RNA synthesis |
|  | AGTTCACCTTGATGCCGTTCTT |  |
| dsGFP-R | AGTTCACCTTGATGCCGTTCTT | double-stranded RNA synthesis |
| EF1-α-F | GGTGCTGGACAAGCTGAAGGC | real-time RT-PCR |
| EF1-α-R | CGTTCCGGTGATCATGTTCTTGATG | real-time RT-PCR |
| *Pm*DOME-F | CTCAGGCTATGTTTCTCAGGATTCA | real-time RT-PCR |
| *Pm*DOME-R | CACGGCAGTTCCTTTATGGTCT | real-time RT-PCR |
| *Pm*JAK-F | TGCTGTTCCGACTGCGTTTC | real-time RT-PCR |
| *Pm*JAK-R | GCGTGGAAGTCTGCTCGAAC | real-time RT-PCR |
| *Pm*STAT-qRT-F | TATATCCGAATGTGCCTAAG | real-time RT-PCR |
| *Pm*STAT-qRT-R | ATAGTTTGTGGTGTGTTGGG | real-time RT-PCR |
| *Pm*SOCS2-qRT-F | CTGCCAAACGCCCACTTC | real-time RT-PCR |
| *Pm*SOCS2-qRT-R | CGTGGCAGGCATTGTGTG | real-time RT-PCR |
| *Pm*Dorsal-qRT-F | TCACTGTTGACCCACCTTAC | real-time RT-PCR |
| *Pm*Dorsal-qRT-R | GGAAAGGGTCCACTCTAATC | real-time RT-PCR |
| *Pm*IMD-F | CGA GAC AAG GTC GAG GTC AG | real-time RT-PCR |
| *Pm*IMD-R | CTC GTA CAC TCG GTC GAC ATT A | real-time RT-PCR |
| *Pm*ProPO2-F | GCCAAGGGGAACGGGTGATG | real-time RT-PCR |
| *Pm*ProPO2-R | TCCCTCATGGCGGTCGAGGT | real-time RT-PCR |
| *Pm*Vago5-F | AGAAGCATTTAGGCTCAGGGCAG | real-time RT-PCR |
| *Pm*Vago5-R | GATGGCCAGAGTTATTGTGACGC | real-time RT-PCR |
| *Pm*ALFPm3-F | CCCACAGTGCCAGGCTCAA | real-time RT-PCR |
| *Pm*ALFPm3-R | TGCTGGCTTCTCCTCTGATG | real-time RT-PCR |
| *Pm*PEN3-F | GGCTTAGCCCCTTACA | real-time RT-PCR |
| *Pm*PEN3-R | GACCCATACCTACAAATAAC | real-time RT-PCR |
| Crustin*Pm*1-F | CTGCTGCGAGTCAAGGTATG | real-time RT-PCR |
| Crustin*Pm*1-R | AGGTACTGGCTGCTCTACTG | real-time RT-PCR |
| Crustin*Pm*7-F | GGCATGGTGGCGTTGTTCCT | real-time RT-PCR |
| Crustin*Pm*7-R | TGTCGGAGCCGAAGCAGTCA | real-time RT-PCR |
| WSSV-VP28-F | AAACCTCCGCATTCCTGTGA | real-time RT-PCR |
| WSSV-VP28-R | TCCGCATCTTCTTCCTTCAT | real-time RT-PCR |
| UPDOME-F1 | CCGGGGAACAGTCGTCGTGAGC | Sequencing |
| DOME-R | TTTTTGCCTTCTGAAGAC | Sequencing |
| Seq_DOME1-F | GGGGAAATACTTGACGGAAA | Sequencing |
| Seq_DOME2-R | CACAATGACACCATTCC | Sequencing |
| Seq_DOME3-F | ATCCACCACAGGATG | Sequencing |
| Seq_DOME4-R | CTCAATTGGGCCAGGAAC | Sequencing |
| Seq_DOME5-F | GAGACAAGCCAGGACAAG | Sequencing |
| Seq_DOME6_R | ACCTACTCTGTTCACTGGTGT | Sequencing |
| Seq_DOME7_R | CCAAGCCAGTGATATTA | Sequencing |
| Seq_DOME8_R | CCCCCACTCTCCACGTGG | Sequencing |
| Seq_DOME9_R | CGGCACAATCACTTATAC | Sequencing |
| Seq_DOME10_F | GCCACCAGACCATAAAGG | Sequencing |
| Seq_DOME11_F | AGCATTGTTAATTGGGGG | Sequencing |

**
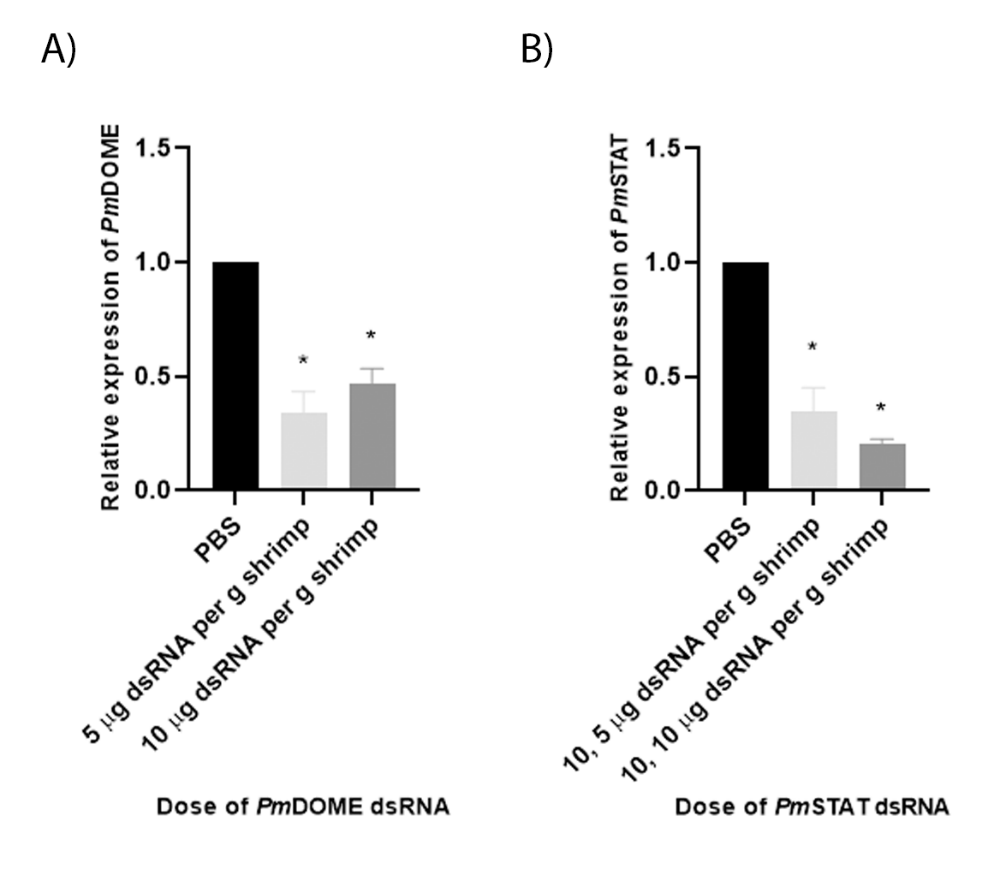
**

**Fig. S1 The silencing efficiency of *Pm*Dome and *Pm*STAT dsRNA injection.** The expression level of *Pm*DOME (A) or *Pm*STAT (B) in shrimp at 24 h post injection with different dosages of dsRNAs. Bars indicate the mean ± SD. Statistical analysis was performed using one-way ANOVA followed by Duncan's new multiple range test. The data were derived from three independently triplicate experiments and considered for statistical differences with the significance at p < 0.05.
